# Fufang Xueshuantong Improves Diabetic Cardiomyopathy by Regulating the Wnt/*β*-Catenin Pathway

**DOI:** 10.1155/2022/3919161

**Published:** 2022-09-19

**Authors:** Meizhong Peng, Hanying Liu, Qingxuan Ji, Pan Ma, Yiting Niu, Shangqiu Ning, Huihui Sun, Xinxin Pang, Yuqian Yang, Yuting Zhang, Jing Han, Gaimei Hao

**Affiliations:** ^1^College of Traditional Chinese Medicine, Beijing University of Chinese Medicine, Beijing, China; ^2^Beijing Anzhen Hospital, Capital Medical University, Beijing, China; ^3^School of Chinese Material Medica, Beijing University of Chinese Medicine, Beijing, China; ^4^Institute of Chinese Medicine, Beijing University of Chinese Medicine, Beijing, China; ^5^Institute of Basic Theory for Chinese Medicine, China Academy of Chinese Medical Sciences, Beijing, China; ^6^Gansu Provincial Hospital of Traditional Chinese Medicine, Gansu, China

## Abstract

Diabetic cardiomyopathy (DCM) is one of the main complications of diabetic patients and the major reason for the high prevalence of heart failure in diabetic patients. Fufang Xueshuantong (FXST) is a traditional Chinese medicine formula commonly used in the treatment of diabetic retinopathy and stable angina pectoris. However, the role of FXST in DCM has not yet been clarified. This study was conducted to investigate the effects of FXST on diabetic myocardial lesions and reveal its molecular mechanism. The rats were intraperitoneally injected with 65 mg/kg streptozotocin (STZ) to induce diabetes mellitus (DM). DM rats were given saline or FXST. The rats in the control group were intraperitoneally injected with an equal amount of sodium citrate buffer and gavaged with saline. After 12 weeks, echocardiography, heart weight index (HWI), and myocardial pathological changes were determined. The expression of transforming growth factor-beta1 (TGF-*β*1), collagen I, and collagen III was examined using immunofluorescence staining and western blot. The expressions of Wnt/*β*-catenin signaling pathway-related proteins and mRNA were detected by western blot and real-time PCR. The results showed that FXST significantly improved cardiac function, ameliorated histopathological changes, and decreased HWI in the DM rats. FXST significantly inhibited the expression of myocardial TGF-*β*1, collagen I, and collagen III in DM rats. Furthermore, FXST significantly inhibited the Wnt/*β*-catenin pathway. Taken together, FXST has a protective effect on DCM, which might be mediated by suppressing the Wnt/*β*-catenin pathway.

## 1. Introduction

Diabetic cardiomyopathy (DCM) is a major cause of the high prevalence of heart failure in diabetic patients [1]. DCM is characterized by specific myocardial structural and functional abnormalities at different stages, which cannot be completely explained by other cardiovascular or noncardiovascular reasons [2]. The main pathological mechanisms of DCM are hyperglycemia, reactive oxygen species, abnormal fatty acid metabolism, microvascular dysfunction, and myocardial fibrosis [3, 4]. At present, the treatment of DCM is still based on antiglycemic agents, combined with angiotensin-converting enzyme inhibitors, diuretics, and *β*-blockers drugs. However, the risk of drug intolerance and electrolyte disorders must be considered [4]. Therefore, further studies are needed to elucidate the pathogenesis of DCM and investigate new therapies.

Myocardial fibrosis is one of the principal pathological features in the late development of DCM [5]. Hyperglycemia usually induces abnormal proliferation of myocardial fibroblasts and deposition of extracellular matrix (ECM) to accelerate the process of myocardial fibrosis, ultimately leading to cardiac insufficiency [6, 7]. It was reported that the Wnt/*β*-catenin signaling pathway can regulate cardiac fibroblast activation and ECM secretion during cardiac fibrosis [8, 9]. In addition, the previous study showed that the protein expression of Wnt2 and *β*-catenin is upregulated in the left ventricles of streptozotocin (STZ)-induced diabetes mellitus (DM) rats, and blocking the Wnt/*β*-catenin signaling pathway can put off the development of DCM [10].

The Fufang Xueshuantong (FXST) formula, currently used in the clinical treatment of diabetic retinopathy and stable angina pectoris, is composed of *Panax notoginseng (Burk.)* F. H. Chen (San-Qi), *Salvia miltiorrhiza Bunge* (Dan-Shen), *Astragalus membranaceus (Fisch.)* Bunge (Huang-Qi) and *Scrophularia ningpoensis Hemsl.* (Xuan-Shen) [11]. In the blood stasis rats established by injection of adrenalin hydrochloride, FXST could reduce blood viscosity and promote microcirculation [12]. In STZ-induced diabetic rats, FXST attenuated diabetic retinopathy by improving the pathological changes in the retina and hemodynamic status [13, 14]. Moreover, FXST moderated kidney hypertrophy and renal histology in the high-fat diet/STZ-induced diabetic nephropathy rats [15]. The clinical experiments demonstrated that FXST can effectively relieve traditional Chinese medicine syndromes of patients with stable angina pectoris of coronary heart disease, can cut down the dosage of nitroglycerin, and has no obvious toxic side effects [16]. But there are few reports about the effects of FXST on DCM. Hence, the purpose of this study was to observe the effects of FXST on DCM and reveal its molecular mechanism, which might provide new options for DCM patients.

## 2. Materials and Methods

### 2.1. Materials and Reagents

Antibodies against transforming growth factor-beta1 (TGF-*β*1) (21898-1-AP), collagen III (22734-1-AP), phospho-GSK-3*β* (Ser 9) (67558-1-Ig), GSK-3*β* (15113-1-AP), *β*-catenin (51067-2-AP), Wnt2 (66656-1-Ig) and c-Myc (10828-1-AP) were purchased from Proteintech Group, Inc (Chicago, USA). Collagen I (AF7001) antibody was obtained from Affinity (USA). WISP1 (ab178547), collagen I (ab255809), collagen III (ab184993), and TGF-*β*1 (ab179695) antibodies were purchased from Abcam (Cambridge Science Park, UK) for western blot analysis. STZ was provided by Sigma-Aldrich (No : S0130-1G; St Louis, USA). The primers of Wnt2, *β*-catenin, c-Myc, GSK-3*β,* and *β*-actin were all entrusted to Sangon Biotech (Shanghai, China) Co. Ltd., for synthesis.

### 2.2. FXST Preparation

The preparation method of FXST was in accordance with the Chinese Pharmacopoeia (2020). *Panax notoginseng (Burk.)* F. H. Chen (250 g) was crushed and extracted twice with 50% ethanol, the filtrate was combined, the ethanol was recovered and concentrated into an ointment, and the dregs were dried and pulverized into fine powder for use. The remaining three herbs such as *Astragalus membranaceus (Fisch.) Bunge* (80 g), *Salvia miltiorrhiza Bunge* (50 g), and *Scrophularia ningpoensis Hemsl.* (80 g) were heated and refluxed with 50% ethanol two times. Then the filtrate was combined, ethanol was recovered and concentrated to an appropriate amount, and finally mixed with the above-prepared ointment and fine powder, dried, crushed, and obtained. The ultra-performance liquid chromatography-mass spectrometry analysis of FXST was determined in our previous work [14].

### 2.3. Animals and Treatment

Male Sprague-Dawley rats weighing 180–200 g were purchased from Vital River Laboratory Animal Technology Co., Ltd. (Beijing, China). The rats were raised in a specific pathogen-free environment, with 12 h light/12 h dark cycles and free access to food and water. All animal experimental protocols were approved by the Ethical Committee of Experimental Animal Care, China Academy of Chinese Medical Sciences, and complied with the National Institutes of Health guidelines (Guide for the care and use of laboratory animals).

After two weeks of adaptive feeding, the rats in the control group (*n* = 8) were intraperitoneally injected with sodium citrate buffer. The remaining rats were injected with an equal volume of 65 mg/kg STZ dissolved in a 0.1 M sodium citrate buffer (pH 4.5) to induce diabetes [17, 18]. Following one week of treatment, blood was collected from the tail vein to measure the blood glucose. If the fasting blood glucose level was higher than or equal to 16.7 mmol/L, the DM model was successfully established. Rats with blood glucose <16.7 mmol/L, were excluded from the study.

The DM rats were randomly divided into 2 groups: the DM group (*n* = 12) and the FXST group (*n* = 12). The rats in the FXST group were orally gavaged with FXST (1.05 g/kg/day) once a day for 12 weeks. The rats in the control and DM groups were orally gavaged with an equal amount of saline. The body weight and blood glucose of the rats were measured at 0, 4, 8, and 12 weeks and 1, 2, 4, 8, and 12 weeks, respectively. At the end of the experiment, the insulin level was measured using enzyme-linked immunosorbent assay analysis according to the manufacturer's instructions (80-INSRT-E01, ALPCO, USA).

### 2.4. Hemodynamic Detection

After 12 weeks of drug intervention, the rats were weighed, anesthetized by inhalation with isoflurane, and fixed in the supine position. A Standard II lead electrocardiogram was monitored. The hemodynamic examination was performed according to the requirements of the MP150 multichannel physiological signal recording and analysis system (Biopac, USA). The right common carotid arteries of the rats were separated, the distal ends were ligated, and the proximal ends were clamped with an arterial clamp. A 20 G vein indwelling needle was inserted through the right carotid artery and connected with the pressure transducer, and then detected by an MP150 multiguide physiological recorder. Cardiac hemodynamic indexes were recorded: heart rate, cardiac index (CI), left ventricular systolic pressure (LVSP), left ventricular end-diastolic pressure (LVEDP), dp/dt min, and dp/dt max.

### 2.5. Echocardiography

The echocardiography was conducted using the Vevo 770 ultrasound imaging system (Visual Sonies, Canada). The rats were anesthetized and fixed, and the left ventricle long and short axis sections were obtained using an RMV-716 probe at a frequency of 17.5 MHz at the left sternal border. Ejection fraction (EF) and fractional shortening (FS) were measured and analyzed. The ultrasonic measurements were averaged over 3 consecutive cardiac cycles. Following the measurements of cardiac function, blood from the abdominal aorta of rats was collected, the whole heart was weighed, and the heart weight index (HWI = heart weight/body weight) was calculated.

### 2.6. Histopathologic Evaluation

The heart tissue was fixed in 4% paraformaldehyde for 48 h, dehydrated by an ethanol gradient, and then embedded in paraffin. Paraffin-embedded heart tissues were sliced into 4 *μ*m sections. Hematoxylin-eosin staining and Masson staining were performed to evaluate histological changes. The morphology of the left ventricle was observed under the microscope. Additionally, the collagen volume fraction was quantitatively analyzed by the Image-Pro-Plus 6.0 Image software.

### 2.7. Immunofluorescence

The sections were routinely deparaffinized to water, followed by antigenic repair, and then nonspecific epitopes were blocked. The slices were incubated with the primary antibody (TGF-*β*1, collagen I, collagen III, 1 : 100 dilution) overnight at 4°C. On the following day, slides were washed with phosphate buffer saline three times and incubated with fluorescein-labeled secondary antibody for 1 h at 37°C. The nuclei were stained with 4′,6-diamidino-2-phenylindole for 5 min. The images were captured by a laser scanning confocal microscope (Leica, SP8, Germany) and analyzed for fluorescence intensity with Image J software.

### 2.8. Western Blot

Prechilled RIPA lysate (p0013 B, Beyotime, Shanghai, China) with a protease inhibitor mixture was added to the myocardial tissues. The tissues were fully ground and lysed. The homogenate was allowed to stand for 10 min on ice and centrifuged at 12,000 r/min for 20 min at 4°C to obtain the protein from the supernatant. The protein concentration was detected with the protein quantification kit (MB155207 A, Pierce, USA). Subsequently, the proteins were separated on a SDS/PAGE gel and transferred to the polyvinylidene fluoride membrane (Millipore, Massachusetts, USA). The membrane was blocked with 5% skim milk and incubated with *β*-actin, TGF-*β*1, collagen I, collagen III, phospho-GSK-3*β*, GSK-3*β*, *β*-catenin, Wnt2, c-Myc, and WISP1 antibodies at 4°C overnight. After being washed with tris-buffered saline and tween 20, the membranes were incubated with goat anti-rabbit IgG or goat anti-mouse IgG (Sc-2004/Sc-2005, Santa Cruz Biotechnology, Inc. USA). *β*-actin served as the internal control. The protein bands were detected using a gel imager (Bio-Rad, USA) and quantified with Quantity One v.4.6.2 software.

### 2.9. Real-Time PCR

Total RNA was extracted from cardiac tissues using Trizol reagent (15596018, Ambion, USA) according to the manufacturer's instructions. The RNA concentration was determined using a NanoDrop ND-2000 spectrophotometer (Thermo Scientific, USA). Then, the RNA was reverse transcribed into cDNA using a reverse transcription kit (Ambion, USA). The real-time polymerase chain reaction was performed with the SYBR Green qPCR Master Mix on an Applied Biosystems Prism 7500 Sequence Detection System. The **β**-actin mRNA expression level was used as the internal control. The 2^−ΔΔCt^ method was used to calculate the expression of the target genes. The PCR primer sequences (5′ ⟶ 3′) were shown as follows:

- *β*-catenin (Forward primer), GGACTCTAGTGCAGCTTCTGGGTTC
- *β*-catenin (Reverse primer), ACAGATGGCAGGCTCGGTAATG
- c-Myc (Forward primer), GCTCTCCGTCCTATGTTGCG
- c-Myc (Reverse primer), TCGGAGACCAGTTTGGCAG
- GSK-3*β* (Forward primer), CGAACTCCACCAGAGGCAATC
- GSK-3*β* (Reverse primer), TGTCCACGGTCTCCAGCATTA
- Wnt2 (Forward primer), GCTGCGAAGTTATGTGTTGTG
- Wnt2 (Reverse primer), GTTGTCCAGTCGGCACTCT
- *β*-actin (Forward primer), GGAGATTACTGCCCTGGCTCCTA
- *β*-actin (Reverse primer), GACTCATCGTACTCCTGCTTGCTG

### 2.10. Statistical Analysis

The data were reported as the mean ± standard deviation (SD). When the data conformed to the normal distribution, one-way analysis of variance (ANOVA) was used for comparison among multiple groups followed by the least significant difference (LSD) test for equal variances or the Games-Howell test for unequal variances; When the data did not conform to the normal distribution, Kruskal-Wallis test was used for comparison among multiple groups (SPSS software 23.0, SPSS Inc., Chicago, IL). *P* < 0.05 was considered statistically significant.

## 3. Results

### 3.1. FXST Increased Insulin Concentration in the DM Rats

**Figure 1:**
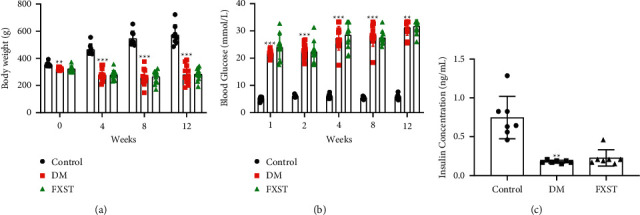
Effect of FXST on general characteristics in DM rats. (a) Body weight was measured at 0, 4, 8, and 12 weeks. (b) Blood glucose was measured at 1, 2, 4, 8, and 12 weeks. (c) Insulin levels were measured at termination. Data are expressed as the mean ± SD (*n* = 7–12 for each group). ^*∗∗*^*P* < 0.01, ^*∗∗∗*^*P* < 0.001 vs. the Control group.

The typical symptoms of STZ-induced type 1 diabetic rats included polydipsia, polyphagia, polyuria, emaciation, hyperglycemia, and low insulin levels [19]. As shown in Figure 1(a), the body weight of the control group rats increased steadily, while the weight of the DM group rats was significantly lower than that of the control group (*P* < 0.01 or 0.001), and there was no significant difference in body weight between the DM and FXST group (*P* > 0.05). Blood glucose was significantly elevated in the DM group as compared to the control group (*P* < 0.01 or 0.001), and FXST treatment had no significant effect on blood glucose in the diabetic rats (*P* > 0.05, Figure 1(b)). In addition, insulin concentration in the DM group was significantly reduced compared to the control group (*P* < 0.01). The insulin concentration of the FXST group was higher than that of the DM group without a statistical difference (*P* > 0.05, Figure 1(c)).

### 3.2. FXST Improved Cardiac Function in the DM Rats

**Figure 2:**
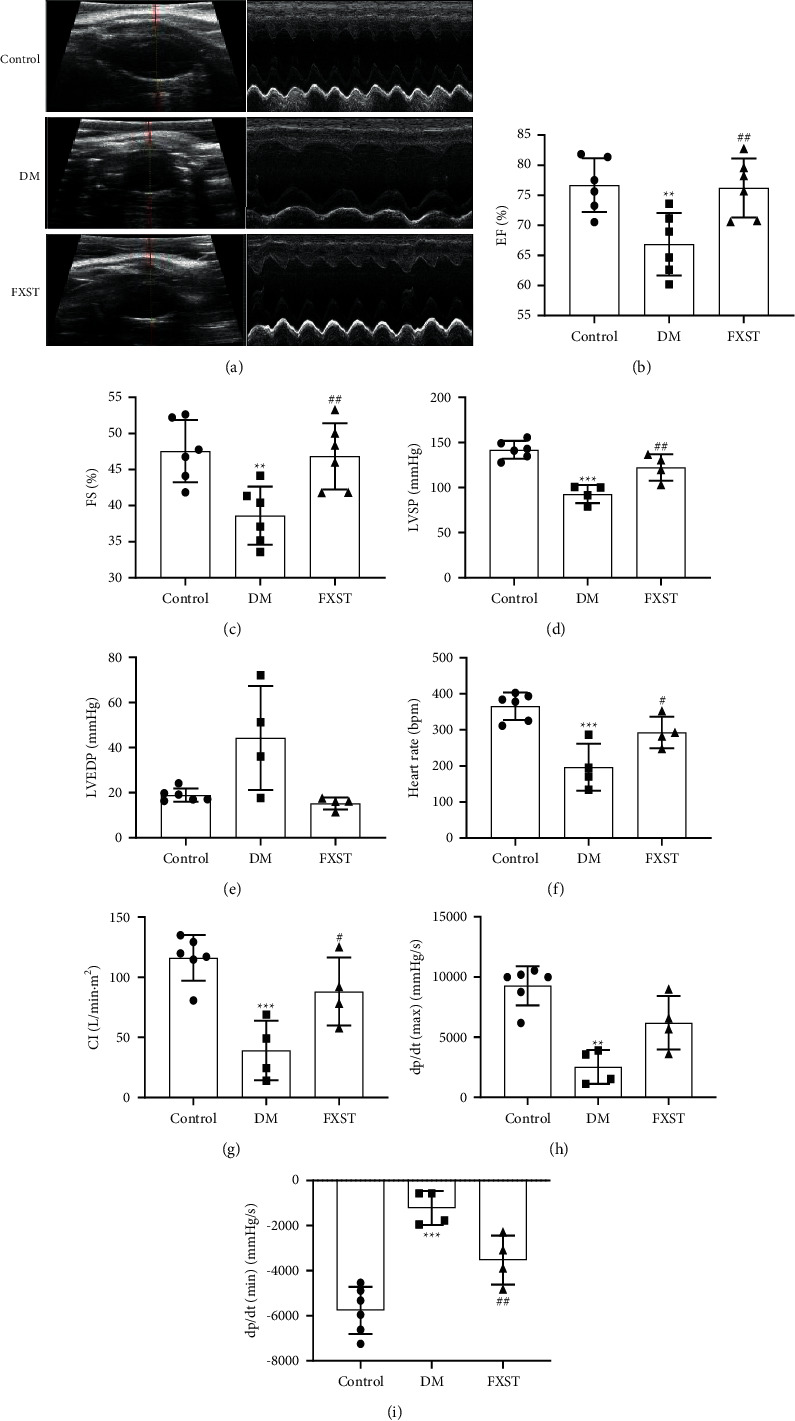
Effects of FXST on cardiac function in DM rats. The model of DCM was induced by intraperitoneal injection of 65 mg/kg STZ and the related parameters were determined by hemodynamic and echocardiography assays 12 weeks later. (a) Representative images of cardiac echocardiogram. (b) EF, (c) FS, (d) LVSP, (e) LVEDP, (f) Heart rate, (g) CI, (h) dp/dt max, and (i) dp/dt min were detected. Data are expressed as the mean ± SD (*n* = 4–6 for each group). ^*∗∗*^*P* < 0.01, ^*∗∗∗*^*P* < 0.001 vs. the Control group, ^#^*P* < 0.05, ^##^*P* < 0.01 vs. the DM group.

The left ventricular hemodynamic and echocardiography parameters were measured to assess the cardiac diastolic and systolic function of the rats. As shown in Figures 2(a)–2(i), compared with the control group, the heart rate, CI, LVSP, dp/dt min, dp/dt max, EF, and FS of the DM group significantly decreased (*P* < 0.01 or 0.001). FXST treatment significantly increased heart rate, CI, LVSP, dp/dt min, EF, and FS (*P* < 0.05 or 0.01). FXST treatment exhibited a trend to enhance dp/dt max (*P* > 0.05). In addition, LVEDP was increased while FXST was decreased in DM rats (*P* > 0.05). These results testified that FXST could alleviate cardiac dysfunction and delay DCM progression in diabetic rats.

### 3.3. FXST Improved Myocardial Histomorphology and Structure in the DM Rats

**Figure 3:**
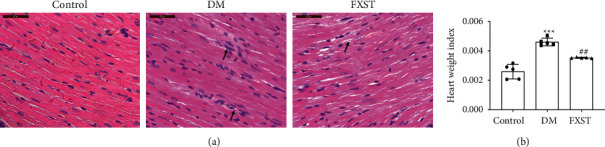
Effects of FXST on myocardial histomorphology and structure in DM rats. (a) Representative images of hematoxylin-eosin staining of myocardial tissues (400×, magnification), arrows represent widened myocardial cell gap and inflammatory cell infiltration. (b) The heart weight index of each group was calculated. Data are expressed as the mean ± SD (*n* = 5 for each group). Scale bar = 50 *μ*m. ^*∗∗∗*^*P* < 0.001 vs. the Control group, ^##^*P* < 0.01 vs. the DM group.

As shown in Figure 3(a), myocardial hematoxylin-eosin staining showed that cardiomyocytes were orderly arranged with uniform nuclear and cytoplasmic staining in the control group. In the DM group, cardiomyocytes were disordered with inflammatory cell infiltration, and uneven nuclear and cytoplasmic staining, which were reversed after FXST intervention. Besides, the effect of FXST was also evaluated by HWI (Figure 3(b)). Compared with the control group, the HWI of the DM group significantly increased (*P* < 0.001), whereas FXST markedly reduced HWI (*P* < 0.01). The results showed that FXST could significantly alleviate the abnormal myocardial tissue morphology and structure of diabetic rats.

### 3.4. FXST Alleviated STZ-Induced Cardiac Fibrosis in the DM Rats

**Figure 4:**
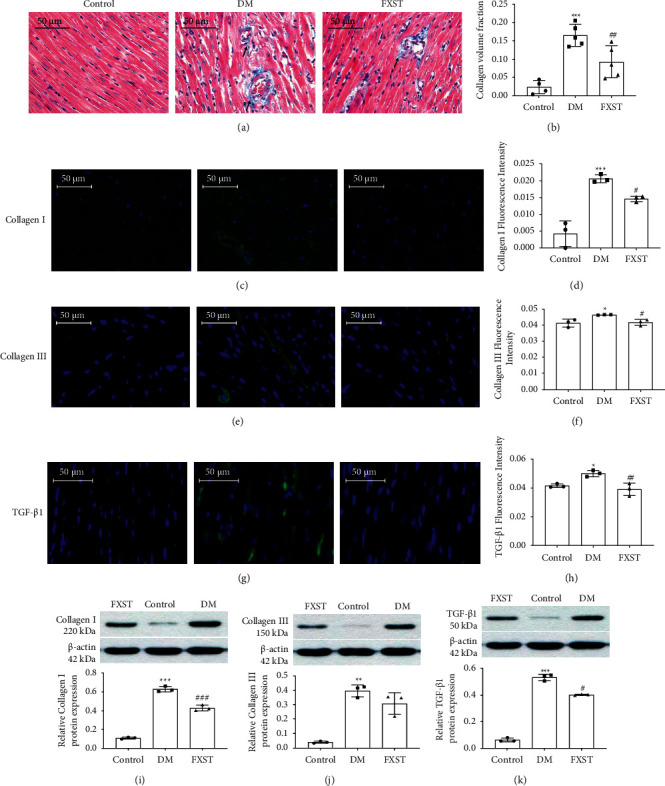
FXST alleviated STZ-induced cardiac fibrosis in DM rats. (a) Representative images of Masson staining of myocardial tissues (400×, magnification). Arrows represent the fibrotic area. (b) The quantification analysis of the collagen volume fraction of myocardial tissues. Representative immunofluorescence images were shown for the expression of collagen I (c), collagen III (e), and TGF-*β*1 (g). The expression level of collagen I (d), collagen III (f), and TGF-*β*1 (h) was detected and quantified. (i–k) Western blot was used to determine the protein expression of collagen I, collagen III, and TGF-*β*1 in the myocardial tissues of rats in each group. Data are expressed as the mean ± SD (*n* = 3–5 for each group). Scale bar = 50 *μ*m. ^*∗*^*P* < 0.05, ^*∗∗*^*P* < 0.01, ^*∗∗∗*^*P* < 0.001 vs. the Control group, ^#^*P* < 0.05, ^##^*P* < 0.01, ^###^*P* < 0.001 vs. the DM group.

The results of Masson staining showed that the deposition of myocardial collagen fibers in the heart tissue of the DM group was significantly higher than that of the control group (*P* < 0.001). Inversely, the deposition area of collagen fibers in the myocardial tissue of the FXST group was significantly reduced compared with the DM group and the degree of fibrosis was reduced (*P* < 0.01, Figures 4(a)-4(b)). We further determined the levels of collagen I, collagen III, and TGF-*β*1 by immunofluorescence and Western blot. The expression levels of collagen I, collagen III, and TGF-*β*1 was significantly elevated in the myocardium of the DM group compared with the control group (*P* < 0.05 or 0.01 or 0.001), nevertheless, FXST downregulated the expression of collagen I, collagen III, and TGF-*β*1 compared to the DM group (*P* < 0.05 or 0.01 or 0.001, Figures 4(c)–4(k)). As consequence, FXST could inhibit the expression of fibrosis-associated genes in DCM.

### 3.5. FXST Attenuated Diabetic Cardiomyopathy via Regulating Wnt/*β*-Catenin Pathway

**Figure 5:**
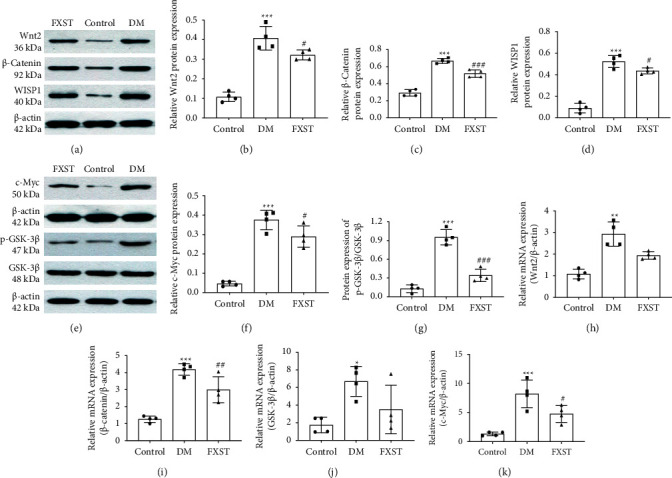
FXST attenuated diabetic cardiomyopathy by regulating the Wnt/*β*-catenin pathway. (a-g) Western blot was used to determine the protein expression of Wnt2, *β*-catenin, WISP1, c-Myc, and p-GSK-3*β* in the myocardial tissues of rats in each group. (h-k) Real-time PCR was performed to measure the mRNA expression of Wnt2, *β*-catenin, GSK-3*β,* and c-Myc in the myocardial tissues of rats in each group. Data are expressed as the mean ± SD (*n* = 4 for each group). ^*∗*^*P* < 0.05, ^*∗∗*^*P* < 0.01, ^*∗∗∗*^*P* < 0.001 vs. the Control group, ^#^*P* < 0.05, ^##^*P* < 0.01, ^###^*P* < 0.001 vs. the DM group.

Western blot demonstrated that compared with the control group, the protein expression levels of Wnt2, *β*-catenin, WISP1, c-Myc, and p-GSK-3*β* in the myocardial tissues of the DM group were significantly upregulated (*P* < 0.001), and downregulated by FXST treatment (*P* < 0.05 or 0.001, Figures 5(a)–5(g)).

The results of the real-time PCR determined that compared to the control group, the myocardial mRNA Wnt2, *β*-catenin, GSK-3*β*, and c-Myc in the DM group were significantly increased (*P* < 0.05 or 0.01 or 0.001), by contrast, the mRNA expression levels of *β*-catenin and c-Myc were significantly decreased in the FXST group compared with the DM group (*P* < 0.05 or 0.01, Figures 5(h)–5(k)).

## 4. Discussion

In the present study, we investigated the effect of FXST in STZ-induced diabetic rats and confirmed the following points. (1) FXST improved cardiac function and histomorphology; (2) FXST significantly alleviated collagen deposition and downregulated TGF-*β*1, collagen I, and collagen III expression; and (3) FXST suppressed the Wnt/*β*-catenin pathway.

### 4.1. FXST Improved the Cardiac Function and Pathology of DM Rats

In the current study, we found that body weight loss, elevated blood glucose levels, HWI, cardiomyocyte disorder, and myocardial fiber gap widening were the main pathological features in STZ-induced diabetic rats. These changes were consistent with the reports of the DCM rat model [19]. It has been reported that FXST improves the hemodynamic status in acute myocardial ischemic canines established by ligating the coronary artery anterior descending branch [20]. Panax notoginseng saponins and *Salvia miltiorrhiza Bunge*, the main components of FXST, increased EF, FS, and LVSP and decreased HWI in the myocardial infarction rats [21, 22]. Moreover, Tanshinone IIA, one of the main monomers of *Salvia miltiorrhiza Bunge*, has been demonstrated to ameliorate DCM [23]. Notoginsenoside R1, a newly extracted phytoestrogen from *Panax notoginseng (Burk.)* F. H. Chen could suppress oxidative stress and cardiac fibrosis to prevent DCM in the db/db mice [24]. The above findings indicated that FXST may become a potential therapeutic drug for DCM. Accordingly, we assessed the pharmacologic effects of FXST in the STZ-induced DCM rats. In this study, we clarified that FXST improved the cardiac function of DCM rats. FXST enhanced EF and FS, maintained hemodynamic stabilization and decreased HWI in the STZ-induced DM rats. Furthermore, the pathological changes of myocardial tissues were also partially restored following FXST treatment. However, we found no significant difference in blood glucose levels between the DM and FXST groups. Based on the concept of “preventive treatment of disease” in Chinese medicine, if diabetic rats are pretreated with FXST in advance, it might play an imperative role in lowering blood glucose and better preventing myocardial injury. Hence, we will investigate whether FXST pretreatment could inhibit the development of STZ-induced diabetes in future studies.

### 4.2. FXST Mitigated Myocardial Fibrosis in the DM Rats

Myocardial fibrosis is a key factor in the deterioration of DCM cardiac function. The characteristic of fibrosis is excessive accumulation of ECM [25], and the fibrin in the ECM is mainly comprised of collagen I and collagen III. Studies have confirmed that the reduction of collagen I and collagen III alleviates myocardial fibrosis in STZ-induced diabetic mice [26, 27]. TGF-*β*1 activation can increase the deposition of ECM in the myocardial interstitium which consequently lead to an increase in the myocardial hardness and a decrease in the ventricular systolic and diastolic function [28]. Our data showed that in comparison with the control group, the protein expression of collagen I, collagen III, and TGF-*β*1 was significantly increased in the DM group, indicating that collagen I, collagen III, and TGF-*β*1 were involved in the progress of DCM. Additionally, studies have determined that tanshinone IIA, astragaloside, and ginsenoside Rb1, which are representative bioactive ingredients of FXST, play a crucial role in inhibiting the development of cardiac fibrosis [29–31]. In our study, compared to the DM group, the protein expressions of TGF-*β*1, collagen I and collagen III were remarkably reduced by FXST. Therefore, we could assume that FXST attenuates the fibrosis process.

### 4.3. FXST Inhibited the Wnt/*β*-Catenin Pathway

The components of the canonical Wnt pathway include ligands (Wnt), transmembrane receptors, cytoplasmic regulatory proteins (GSK-3*β*, *β*-catenin), and nuclear transcription factors. Under myocardial injury conditions, the Wnt pathway is activated. Wnt protein binds to cell surface receptors, resulting in the depolymerization of the GSK-3*β*/APC/Axin degradation complex, which inhibits the phosphorylation of *β*-catenin by GSK-3*β*. Subsequently, *β*-catenin is accumulated in the cytoplasm and then transferred to the nucleus, thus turning on the transcription of downstream target genes (c-Myc, WISP1) [32, 33]. The Wnt/*β*-catenin pathway exerts an imperative role in the regulation of DCM [34] and myocardial fibrosis [9]. *β*-catenin protein expression is usually elevated in various cardiac fibrotic lesions [35]. GSK-3*β* can regulate cardiac hypertrophy and cardiac fibrosis [36]. Under stimulation factors such as ischemia and hypoxia, the dysregulation of c-Myc expression causes fibroblast proliferation and the increase of collagen synthesis, leading to myocardial fibrosis [37]. WISP1 is a member of the connective tissue growth factor family, which promotes proliferation and fibrosis [38]. In our study, the gene and protein expression levels of *β*-catenin, WISP1, Wnt2, c-Myc, and GSK-3*β* in the myocardial tissues of the DM group were increased compared to those of the control group. Besides, it was reported that the downregulation of Wnt2, GSK-3*β,* and *β*-catenin expression may ameliorate cardiac fibrosis in diabetic mice and type 1 diabetic rat [39, 40]. Studies have shown that the effective ingredients of FXST, tanshinone IIA, and ginsenoside Rb1, can inhibit Wnt and *β*-catenin levels in hyperglycemic circumstances [41, 42]. In line with the literature, we found the expressions of *β*-catenin, WISP1, p-GSK-3*β*, Wnt2, and c-Myc in the FXST group were significantly inhibited. It is suggested that the therapeutic effect of FXST on DCM cardiac fibrosis is at least partially attributed to the inhibition of the Wnt/*β*-catenin pathway. However, in future studies, we intend to use Wnt inhibitors or Wnt transgenic mice to more directly demonstrate the cardioprotective effect of FXST through the Wnt/*β*-catenin pathway.

## 5. Conclusion

In summary, FXST could enhance cardiac function and reduce myocardial damage caused by diabetes, and its antimyocardial fibrosis mechanism might be related to the downregulation of the Wnt/*β*-catenin signaling pathway. These findings provide an experimental basis for understanding the beneficial role of FXST in the treatment of DCM. The components of FXST are pretty complex, therefore further studies on the active ingredients of FXST should be carried out. In addition, in-depth research can be conducted to explore the components of FXST that can act on fibrosis and the Wnt pathway.

## Data Availability

All data used to support the findings of this study are available from the corresponding author upon request.
